# Chitosan–Cellulose Multifunctional Hydrogel Beads: Design, Characterization and Evaluation of Cytocompatibility with Breast Adenocarcinoma and Osteoblast Cells

**DOI:** 10.3390/bioengineering5010003

**Published:** 2018-01-09

**Authors:** Poonam Trivedi, Tiina Saloranta-Simell, Uroš Maver, Lidija Gradišnik, Neeraj Prabhakar, Jan-Henrik Smått, Tamilselvan Mohan, Martin Gericke, Thomas Heinze, Pedro Fardim

**Affiliations:** 1 Laboratory of Fibre and Cellulose, Åbo Akademi University, 20500 Turku, Finland; ptrivedi@abo.fi; 2 Johan Gadolin Process Chemistry Centre, Laboratory of Organic Chemistry, Åbo Akademi University, 20500 Turku, Finland; tiina.saloranta-simell@abo.fi; 3 Faculty of Medicine, University of Maribor, 2000 Maribor, Slovenia; uros.maver@um.si (U.M.); lidija.gradisnik@um.si (L.G.); 4 Pharmaceutical Sciences Laboratory, Åbo Akademi University, 20500 Turku, Finland; Neeraj.Prabhakar@abo.fi; 5 Laboratory of Physical Chemistry and Center for Functional Materials, Åbo Akademi University, 20500 Turku, Finland; Jan-Henrik.Smatt@abo.fi; 6 Institute of Chemistry, Karl-Franzens-University Graz, Heinrichstraße 28, 8010 Graz, Austria; tamilselvan.mohan@gmail.com; 7 Institute of Organic Chemistry and Macromolecular Chemistry, Centre of Excellence for Polysaccharide Research, Friedrich Schiller University of Jena, Humboldtstraße 10, D-07743 Jena, Germany; martin.gericke@uni-jena.de; 8 Department of Chemical Engineering, KU Leuven, Celestijnenlaan 200F, B-3001 Leuven, Belgium; thomas.heinze@uni-jena.de

**Keywords:** chitosan, cellulose, coagulation, hydrogel, scaffolds, cytocompatibility, tissue engineering

## Abstract

Cytocompatible polysaccharide-based functional scaffolds are potential extracellular matrix candidates for soft and hard tissue engineering. This paper describes a facile approach to design cytocompatible, non-toxic, and multifunctional chitosan-cellulose based hydrogel beads utilising polysaccharide dissolution in sodium hydroxide-urea-water solvent system and coagulation under three different acidic conditions, namely 2 M acetic acid, 2 M hydrochloric acid, and 2 M sulfuric acid. The effect of coagulating medium on the final chemical composition of the hydrogel beads is investigated by spectroscopic techniques (ATR–FTIR, Raman, NMR), and elemental analysis. The beads coagulated in 2 M acetic acid displayed an unchanged chitosan composition with free amino groups, while the beads coagulated in 2 M hydrochloric and sulfuric acid showed protonation of amino groups and ionic interaction with the counterions. The ultrastructural morphological study of lyophilized beads showed that increased chitosan content enhanced the porosity of the hydrogel beads. Furthermore, cytocompatibility evaluation of the hydrogel beads with human breast adenocarcinoma cells (soft tissue) showed that the beads coagulated in 2 M acetic acid are the most suitable for this type of cells in comparison to other coagulating systems. The acetic acid fabricated hydrogel beads also support osteoblast growth and adhesion over 192 h. Thus, in future, these hydrogel beads can be tested in the in vitro studies related to breast cancer and for bone regeneration.

## 1. Introduction

Polymers, derived from plant and animal sources, such as cellulose and chitosan, have functional groups that provide the unique characteristics, physicochemical properties, and are responsible for their cytocompatibility [1]. Chitosan is the glucosamine polymer obtained after deacetylation of chitin, which results in free amino groups along with abundant hydroxyl groups [2]. Cellulose, on the other hand, possesses already in its main structure abundant hydroxyl groups, leading to extensive hydrogen bonding and stereoregularity in the polymeric chain [3]. Both mentioned naturally derived polymers are non-toxic and cytocompatible [4]. Chitosan and cellulose had been previously successfully processed in the form of beads, fibres, films, nanoparticles, hydrogels, cryogels, and are used in various pharmaceutical and tissue engineering applications [5,6].

Scaffolds prepared from various biomaterials have been used in hard (bone) and soft tissue (skin) engineering [7]. The key requirements for such biomaterial-based scaffolds are cytocompatibility, potential biodegradability, nontoxicity of degradation products, and porosity that suits the chosen cell type [8]. All of these factors altogether contribute to mimicry of the role of the extracellular matrix, by which the scaffolds act as an adhesive substrate, support for cell survival, aids cell migration, and promotes the preservation of the desired cell type [9,10].

Chitosan-based composite hydrogels, such as chitosan/alginate and chitosan/silica, have been tested to promote bone regeneration [11,12]. Similarly, Chitosan-based sponges prepared by lyophilization of chitosan hydrogels, have also been proven to be effective as a bone tissue engineering material [13]. In recent studies, the preparation of highly porous scaffolds from nano-fibrillated cellulose was also investigated [14,15]. The method of designing a biomaterial scaffold has a direct impact on its application in tissue engineering. Thus, when considering the method of preparation of chitosan-cellulose composite hydrogels, these can be classified as physical or chemical hydrogels. In physical hydrogels, the interactions between polymeric chains are van der Waals forces, chain entanglements, hydrogen bonding, and hydrophobic or electronic associations. In the case of chemical hydrogels, the interpolymer interactions are due to crosslinking agents, such as glutaraldehyde, genipin, and tripolyphosphate [16,17,18,19,20,21]. A related well-known fact about chitosan is that its glucosamine residues protonate in diluted acidic solutions and form ionic complexes with anionic species [22,23]. 

In case of chitosan-cellulose-based hydrogel beads design, the solvent system plays a key role in the resulting properties such as chemical constitution, porosity, size, water retention capacity, which ultimately governs the application. Until now acetic acid, *N*-methylmorpholine-*N*-oxide (NNMO), ionic liquids, and NaOH/urea/water solvent system are used to design chitosan-cellulose hydrogel beads of varying properties [24]. Nevertheless, the hydrogel beads previously developed had been tested for different applications, like the adsorption and removal of metal ions, such as copper, iron, and nickel [25,26,27].

We aimed to design chitosan-cellulose hydrogel beads in an environment-friendly medium with variable ionic interactions of the protonated amino groups with the counter ions generated from the coagulating solvent systems. The cytocompatibility of novel materials to be used in biomedical applications is considered as the most crucial initial requirement for their potential application. Therefore, an effort was put to evaluate the same in our study. For this purpose, the novel hydrogel beads were tested towards human breast adenocarcinoma (MDA-MB-231) cells as an example of a soft tissue and towards human osteoblast cells as an example of hard tissue. Initially, we tested the basic cytocompatibility of the novel hydrogel beads, while based on the positive results, additional attachment testing was performed in the case of the osteoblast cells. Finally, a multi-day exposure of the cells to the developed materials was performed to evaluate their potential for tissue engineering applications.

In a previous study, the NaOH/urea/water solvent system was used to prepare the chitosan-cellulose composite solution by freeze-thaw cycles at low temperatures [28]. Thus, we choose the 7% NaOH/12% urea/ 81% water solvent system to prepare the polymer blends. The polymer solution was coagulated in the form of hydrogel beads in 2 M acetic acid, hydrochloric acid, and sulfuric acid, respectively. The effect of polymer concentration and the nature of coagulating acid on the hydrogel beads were investigated by Nuclear Magnetic Resonance (NMR), Infrared (IR) and Raman spectroscopy as well as by X-ray Diffraction (XRD) and Scanning Electron Microscopy (SEM) techniques. These hydrogel beads could be easily transformed into cryogels via lyophilization technique. The designed hydrogel beads coagulated in 2 M acetic acid showed the optimal cytocompatibility and could be used in the future for bone tissue engineering studies that are related to the treatment of bone injuries.

## 2. Materials and Methods

### 2.1. Materials

Enoalfa cellulose pulp with alpha cellulose content >93.5% was provided by Enocell pulp mill, Finland. Low molecular weight chitosan (190–310 kDa with 75–85% degree of deacetylation) was purchased from Aldrich, sodium hydroxide (NaOH, 97% purity) was purchased from Fluka. Urea (CO(NH_2_)_2_, 99.5% purity), sulfuric acid (H_2_SO_4_, 98%), acetic acid (CH_3_COOH, 98%), and hydrochloric acid (HCl, 37%) were obtained from Merck. Ethanol (92%) was purchased from VWR. Deionized water was obtained from the Milli-Q system (0.2 µm filters). MDA-MB-231 cells (Human breast adenocarcinoma), Dulbecco’s modified Eagle’s medium (DMEM) supplemented with 10% fetal bovine serum, 2 mM l-glutamine, and 1% penicillin, streptomycin (*v*/*v*) were purchased from Life Technologies, ThermoFisher Scientific Inc., Darmstadt Germany. WST-1 cell proliferation reagent was from Roche Diagnostics, Germany. 10% DMSO, Human bone osteoblasts (ATCC CRL-11372), DMEM with 5 wt.% FBS were purchased from Life Technologies, ThermoFisher Scientific Inc., Darmstadt, Germany and 3-(4,5-dimethylthiazol-2-yl)-2,5-diphenyltetrazolium bromide (MTT) assay was purchased from Sigma-Aldrich, Hamburg, Germany.

### 2.2. Methods

#### 2.2.1. Preparation of Chitosan-Cellulose Hydrogel Beads

To prepare chitosan–cellulose hydrogel beads, varying proportions of chitosan (10–90%) were mixed with HyCelSol [29] treated cellulose pulp keeping 5% final polymer concentration in the solution. The polymer mixture was added to the NaOH/urea/water (7/11/81) solvent system and stirred for 1 h at room temperature, followed by cooling at −13 °C for 1 h. The obtained composite solution was extruded through a 0.8 mm needle in the form of beads into 250 mL 2 M acetic acid, hydrochloric acid, and sulfuric acid solutions, respectively. The beads formed were kept in the coagulation medium for 2 h, followed by subsequent washing with deionized water until neutral conditions were achieved. The beads extruded in 2 M acetic acid, hydrochloric acid, and sulfuric acids were named as **A**, **B**, and **C** type with the initial chitosan percentage in the blend, respectively. (For example **0A**, **0B** and **0C** had 0% initial chitosan concentration). The pH value of coagulating medium was recorded after hydrogel beads gelation of the **70A, 70B,** and **70C** (hydrogel beads coagulated in 2 M acetic, hydrochloric, and sulphuric acid, respectively with initial chitosan concentration of 70%) samples. Finally, the hydrogel beads were stored in deionized water.

#### 2.2.2. Ftir and Raman Spectroscopy

Nicolet iS 50 FT-IR spectrometer with Raman module from Thermo Scientific (Darmstadt, Germany) was used for spectrometric measurements. FT-IR spectra were collected using tungsten-halogen source and DLaTGS-KBr detector splitter setup with 4.00 cm^−1^ resolution and 64 scans. Raman spectra were collected using a diode laser (power–0.5 W). The detector was InGaAs with CaF_2_ splitter, resolution 8.0 cm^−1^, aperture size 200, and number of scans 64,000. Lyophilized hydrogel beads were cross-sectioned and were analysed.

#### 2.2.3. Solid State ^13^C and ^15^N NMR Spectroscopy

The solid-state ^13^C and ^15^N NMR spectra were recorded with 400 MHz Bruker AVANCE-III NMR spectrometer, equipped with a 4 mm Cross-Polarisation Magic Angle Spinning CP-MAS probe operating frequencies 100.52 MHz (^13^C) and 40.51 MHz (^15^N). The spectra were recorded at the spinning rate of 5 kHz and a contact time of 2 ms (^13^C) and 3.5 ms (^15^N).

#### 2.2.4. Elemental Analysis

A VARIO EL III CHNS analyser (Elementar Analysensysteme GmbH, Langenselbold, Germany) was used for elemental analysis according to standardised procedures. The chlorine content was determined subsequently by combustion of the organic samples and potentiometric titration with AgNO_3_ using a chloride-sensitive electrode.

#### 2.2.5. XRD Analysis

XRD measurements were performed on a Bruker D8 Discover instrument (Bruker, Karlsruhe, Germany) with a Cu K_α_ X-ray source and an HI-STAR area detector. The incident angle was kept at 6°, while the detector angle (2θ) was 25°.

#### 2.2.6. Scanning Electron Microscopy-Energy Dispersive Spectroscopy

The qualitative composition analysis of beads was performed with JEOL JSM-6335F (JEOL Ltd., Tokyo, Japan) with accelerating voltage 15 kV, Working Distance (WD) = 15 mm. Air dried cross-sectioned beads were moulded into the epoxy resin in a gelatinous capsule. The resin was allowed to cure at RT for 24 h. Trimming was performed with a microtome. After trimming, the sample was sputtered with platinum and a fresh surface exposed by slicing a thin layer off of the surface with the microtome. A spot analysis was taken with a COMPO image detector from two places representing different phases of the sample denoted as shades of lighter and darker grey.

The cross-section analysis of beads was performed with LEO Gemini 1530. A Thermo Scientific Ultra Silicon Drift Detector (SDD) equipped with secondary electron backscattered electron and In lens, detector was used. The chitosan-cellulose hydrogel beads were frozen in liquid nitrogen and were lyophilized. The beads were further cross-sectioned, coated with carbon and analyzed. The magnification of the images corresponds to a Polaroid 545 print with the image size of 8.9 × 11.4 cm^2^.

#### 2.2.7. Cytocompatibility Evaluation of Chitosan-Cellulose Hydrogel Beads Coagulated in Different Acids with MDA-MB-231 (Human Breast Adenocarcinoma) Cells

The cell studies were performed under standard conditions in a humidified, 37 °C, 5 wt.% CO_2_ environment using MDA-MB-231 cells. MDA-MB-231 cells (Human breast adenocarcinoma) were cultured in Dulbecco’s modified Eagle’s medium (DMEM) supplemented with 10 wt.% fetal bovine serum (FBS), 2 mM l-glutamine, and 1% penicillin–streptomycin (*v*/*v*). **0A**, **70A**, **0B**, **70B**, and **0C**, **70C**, hydrogel beads that were previously soaked in cell culture medium were placed in a 96 well cell culture plate. MDA-MB-231 cells were added to each type of hydrogel beads for evaluation of attachment and proliferation for 48 h. After incubating the cells with hydrogel beads for 48 h at 37 °C, 5 wt.% CO_2_, 10 µL of WST-1 cell proliferation reagent (Roche Diagnostics, Mannheim, Germany) was added to the respective wells containing 100 µL of cell growth media, and the plate was again allowed to incubate for 3 h at 37 °C, 5 wt.% CO_2_. After the incubation period, the absorbance was read at 430 nm by Tecan Ultra microplate reader (MTX Lab Systems, Inc.) from Bradenton, FL, USA. The number of viable cells was correlated with the observed absorbance from each sample. 10 wt.% DMSO (toxin) was added as a negative control, whereas wells without beads (tissue culture plastic (TCP)) were used as positive control cells and were allowed to grow without any cellulose beads.

#### 2.2.8. Cytocompatibility Evaluation of Chitosan-Cellulose Hydrogel Beads Coagulated in 2 M Acetic Acid with Osteoblast Cells

The **0A**, **50A,** and **70A** hydrogel bead samples suspended in ultrapure water obtained from Milli-Q were used for the osteoblast cells adhesion and proliferation studies. The number of hydrogel beads used was three **0A** and two **50A** and **70A** respectively. The beads were sterilised using UV light (for 30 min) followed by soaking in 1 mL of the cell culture medium (advanced DMEM) with 5 wt.% FBS for 30 min. Human bone osteoblasts (ATCC CRL-11372), were seeded on a P96 well microtiter plate at a concentration of 20.000 cells/cm^2^. Allowing the cells to attach for 24 h, the hydrogel beads samples were added in four repetitions. For this purpose, we used the as-prepared samples, and their dilutions of 1:2, 1:4, and 1:8 in Advanced DMEM with 5 wt.% FBS. After one, four, six, and 192 h of incubation at 37 °C and 5 wt.% CO_2_, the sample cytocompatibility/cytotoxicity was assessed using the MTT assay (Sigma-Aldrich, Germany). The media was changed every other day. Cell viability was determined via the reduction reaction of the tetrazolium salt MTT (3(4,5-dimethylthiazolyl-2)-2,5-diphenyltetrazolium bromide), as determined by measuring the absorbance (using Varioskan, Thermo Fisher Scientific Inc., Germany) at 570 nm [30].

#### 2.2.9. Cell Attachment Testing of Chitosan-Cellulose Hydrogel Beads Coagulated in 2 M Acetic Acid with Osteoblast Cells

The **0A**, **50A,** and **70A** hydrogel bead samples were stored in ultrapure water obtained from Milli-Q. Four beads of sample **70A**, and five beads of samples **0A** and **50A** were poured with 1 mL of Advanced DMEM medium with 5 wt.% FBS and left to culture with it for 30 min. Since the beads were of different sizes and had therefore different weights, as well as different surface to volume rations, our experiments were based on using same weights of the beads instead of the same number. Consequently, the number of beads for respective samples used for the experiments is different. The samples were transferred to a chamber with eight wells (8 well glass slide, slide Chamber, Nunc, Thermo Fisher Scientific, USA) and combined in the cell suspension with a cell density of 17,500 cells/well. The same cell density was used for the control well (as the control we used Advanced DMEM medium with 5 wt.% FBS without beads in tissue culture plastic plates (TCP)). Samples were prepared in duplicates. The obtained cells with beads were cultured at 37 °C, using an atmosphere of 5 wt.% CO_2_. The growth medium was replaced after 72 h cells seeding. After 192 h the samples were stained using the crystal violet dye (0.1 wt.% crystal violet in 20 wt.% ethanol). For this purpose, the medium was pipetted from respective wells, followed by washing of the samples twice with PBS (phosphate buffered saline, Sigma, Darmstadt, Germany), poured with 300 µL of the as-prepared dye solution and left for 5 min at room temperature. The dye was consequently pipetted away, and the samples were washed three times with ultrapure water obtained from Milli-Q system. This was followed by observing the surface of the beads with a Leica DM2500 microscope (Leica, Germany).

#### 2.3.10. Statistical Analysis

The data obtained for both the cell-based tests is presented as mean values ± standard deviation of the mean. Statistical analysis of the significance for the cytocompatibility testing on both cell types was performed using a one-way analysis of variance (ANOVA test) against respective control samples. A *p* < 0.05 (and lower) was considered as significant. All of the statistical tests were performed using Excel 2016.

## 3. Results and Discussions

### 3.1. Effect of Coagulating Medium on the Mechanism of the Chitosan-Cellulose Hydrogel Beads Formation

**Figure 1:**
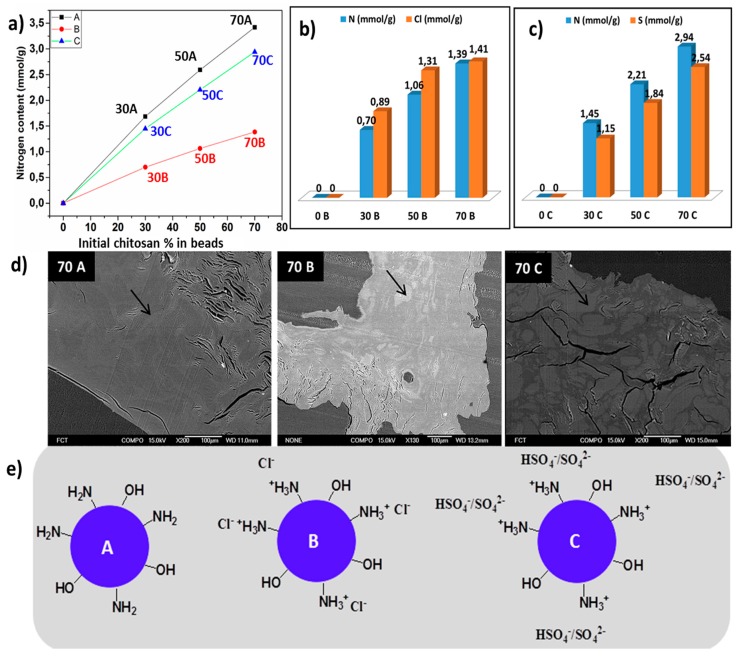
(**a**) Total nitrogen content present in beads with varying chitosan composition coagulated in 2 M acetic acid (**A**), 2 M hydrochloric acid (**B**) and 2 M sulfuric acid (**C**). (**b**,**c**) A comparison of chlorine and sulphur with the nitrogen content in **B** and **C** type. (**d**) Nitrogen element distribution measured via spot analysis in **70A**, **70B**, and **70C** and (**e**) Presentation of possible ionic interactions in **A**, **B** and **C** type.

Chitosan and cellulose were blended in the ratio of 0/100, 10/90, 30/70, 50/50, 70/30, 90/10 in NaOH/urea/water (7/12/81) solvent system. The beads were extruded in 2 M acetic acid, hydrochloric acid, and sulfuric acid, and named as **A**, **B**, and **C**, respectively (Figure 1). The hydrogel beads with a chitosan-cellulose ratio up to 70/30 were stable, and further increasing chitosan content to 90/10 did not result in stable entities. Thus 90/10 beads were not characterized. The chosen coagulating acidic medium affected the final composition of the resulting hydrogel beads. The measured pH value of the coagulating medium after hydrogel formation was 5.0, 1.0, and <1.0 in case of **A**, **B** and **C** systems, respectively.

Further elemental analysis of the samples was performed to understand the mechanism of hydrogel formation in each type of acidic medium. A detailed elemental composition analysis of the dried beads coagulated in 2 M acetic acid (**A**) type, hydrochloric (**B**) type, and sulfuric acid (**C**) type, respectively shows an increasing trend in the nitrogen content, which corresponds to the chitosan content in hydrogel beads from initial 0 to 70% composition. The **70A** has the highest nitrogen content equivalent to 3.4 mmol/g, while **70B** has 1.2 mmol/g and **70C** 2.9 mmol/g, respectively. The beads coagulated in 2 M hydrochloric acid had the lowest nitrogen content in comparison to the two other systems (Figure 1a). The beads coagulated in hydrochloric and sulfuric acid also showed the presence of chlorine and sulphur atoms, respectively (Figure 1b,c). In the case of beads coagulated in 2 M hydrochloric acid and 2 M sulfuric acid, the amino group of chitosan were protonated (NH_3_^+^) and had ionic interactions with the chloride (Cl^−^) and hydrogen sulphate (HSO_4_^−^) or sulphate (SO_4_^2−^) ions. The qualitative analysis of the beads via SEM-EDS displayed shades of lighter and darker grey corresponding to different positions in the bead samples, which has been indicated by arrow in (Figure 1d). Spot analysis of **70A** revealed a homogeneous distribution of chitosan, while in the case of **70B** and **70C**, a non-homogenous chitosan composition was observed. The EDS spectra are included in (Supplementary Figure S1). The elucidated chemistry of the designed beads has been proposed, as shown in Figure 1e. The presence of higher chitosan content in **A**-type of hydrogel beads can be explained by the fact that at pH value of 5, chitosan exhibits reduced solubility, resulting in an irreversible interaction with the cellulose and the enhanced chitosan deposition [31].

In the case of **B**-type, the final coagulating medium had a pH value of 1. Even at low concentrations, HCl is a strong acidic solvent for the dissolution of chitosan. Therefore the maximum dissolution of chitosan in the beads is presumed. In **C**-type, the pH value was <1.0, and sulfuric acid does not lead to the dissolution of chitosan even after the protonation of amino groups (NH_3_^+^). It could be explained by the study by Cui et al., which showed that in the presence of sulfuric acid, the amino groups of chitosan are protonated (NH_3_^+^), but are in strong electrostatic interaction with the sulfonate (SO_4_^2−^) anions [32]. Therefore, the coagulating medium dictates the final chemistry and composition of the chitosan-cellulose hydrogel beads.

### 3.2. Attenuated Total Reflectance–Fourier Transform Infra-Red (ATR–FTIR) and Raman Spectroscopic Analysis

**Figure 2:**
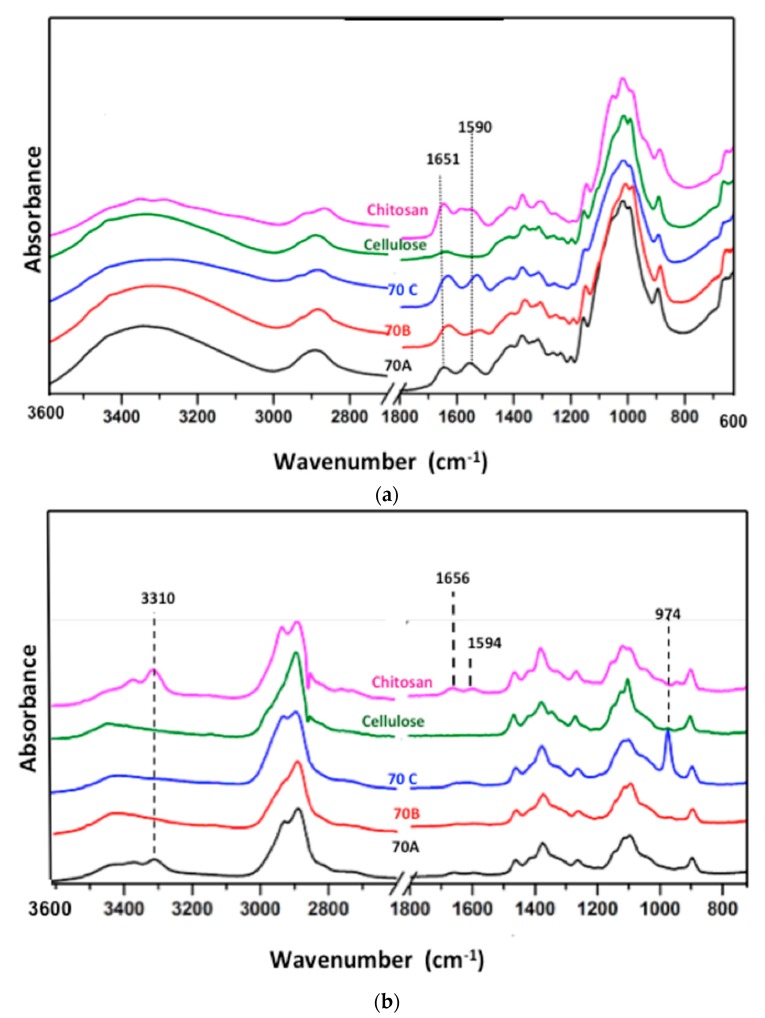
(**a**) Attenuated Total Reflectance–Fourier Transform Infra-Red (ATR-FTIR) and (**b**) Raman spectra of chitosan cellulose and **70A**, **70B** and **70C** lyophilized hydrogel beads.

In the IR spectra of pristine chitosan and cellulose, broad bands are visible in the region 3700–3000 cm^−1^, corresponding to O–H stretching and N–H vibrations. The presence of amide I band at 1651 cm^−1^ due to the carbonyl stretching vibrations, and a bifurcated band with peaks at 1590 cm^−1^ due to amide II, corresponding to N–H bending vibrations and at 1560 cm^−1^, due to free NH_2_ bending vibrations are present in chitosan. A slight variation in the CHx deformations region at 1425 and 1374 cm^−1^, C–O–C and C–O stretching vibration region at 1200–950 cm^−1^ are also apparent (Figure 2a). The chitosan-cellulose hydrogel beads coagulated in 2 M acetic, hydrochloric and sulfuric acid displayed significant variations in the region 4000–1450 cm^−1^ and in the fingerprint region 1450–500 cm^−1^ when compared to the pristine chitosan and cellulose biopolymers. In chitosan^-^cellulose beads, the 3600–3000 cm^−1^ region in **70C** is broader than the **70A** and **70B**. In the 1700–1500 cm^−1^ region corresponding to amide and amino group vibrations, significant variations are observed. In **70A**, **70B**, and **70C**, the peak due to amide I (at 1641 cm^−1^) overlaps with the water absorbance peak. Also, in the band corresponding to free amino groups clear variations are observed. In **70A**, the band appears in the chitosan in the same region at 1555 cm^−1^, while in **70B**, a band with lower intensity exhibiting a slight shift towards right at 1525 cm^−1^ is visible. In the case of **70C** beads, a peak at 1532 cm^−1^ of equal intensity exhibiting a small shift towards the right, is present. The shift in peaks towards the right in the case of **70B** and **70C** is presumably related to the protonated amino (NH_3_^+^) groups bending vibrations having interaction with the counterions [33]. A slight variation in the CHx deformations that correspond to peaks at 1425 and 1374 cm^−1^ can also be observed (Figure 2a).

Similarly, in the region 3600–3100 cm^−1^ of the Raman spectra, the sharp O-H and N-H stretching vibrations in chitosan and broad O–H stretching vibrations in cellulose are visible. In chitosan, the peak due to alkyl groups shows slight bifurcation, which is not apparent in cellulose (Figure 2b). In **70A**, the 3600–3100 cm^−1^ region matches with that of chitosan, and as such, displaying sharp peaks, whereas **70B** and **70C** show broader bands in the same region. The amide and the amino region in **70A** matches with the natural chitosan, indicating the presence of unchanged chitosan. A comparatively less intensity band in **70B** and broadband in **70C** is evident. The band broadening in **70C** could be due to strong ionic interactions between the protonated amino groups and hydrogen sulphate or sulfonate anion. The appearance of a new distinct peak at 974 cm^−1^ in **70C** is due to the presence of SO_4_^2−^ ions [33]. The effect of sulfuric acid has also been explained by the fact that first, the sulfuric acid protonates the amino group, and then acts as a crosslinking agent between the protonated amino groups by sulfate (SO_4_^2−^) ions [33]. Hence, the spectroscopic data confirms the protonation of amino groups and interaction between the amino groups and negatively charged species in case of **70B** and **70C** hydrogel beads, while in **70A** beads, no such evidence was observed.

### 3.3. Solid-State ^13^C and ^15^N Nuclear Magnetic Resonance

**Figure 3:**
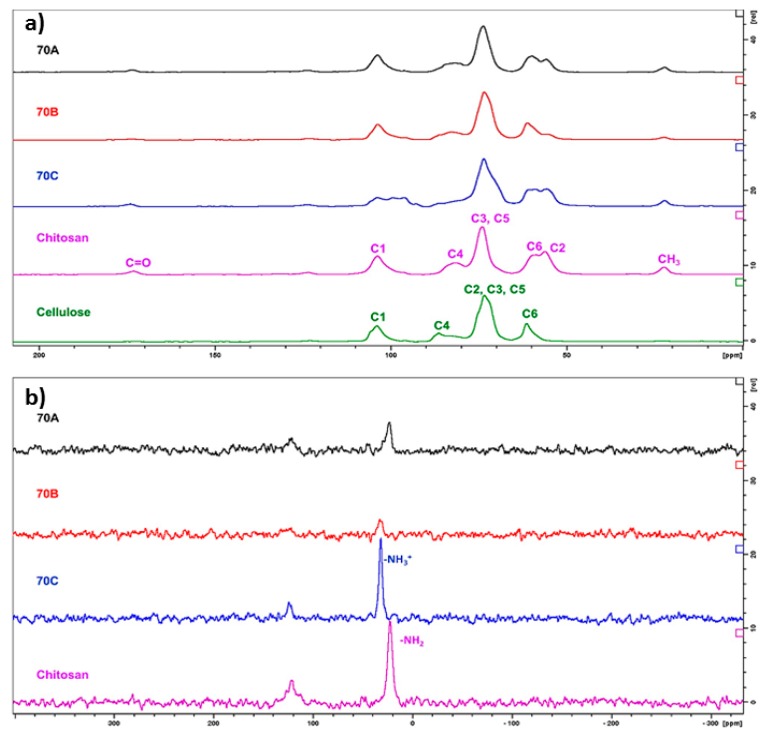
(**a**) CP-MAS ^13^C NMR spectra (**b**) CP-MAS ^15^N NMR spectra of chitosan, cellulose, **70A**, **70B,** and **70C** chitosan-cellulose lyophilized hydrogel beads.

In order to study the hydrogel beads further, chitosan, cellulose, H_2_SO_4_ treated chitosan reference samples, as well as the lyophilized hydrogel beads **70A**, **70B**, and **70C,** were analysed by solid-state CP-MAS ^13^C- and ^15^N NMR spectroscopy. The characteristic signals observed in the CP-MAS ^13^C NMR spectra of cellulose and chitosan (Figure 3a) have been reported in the literature [34,35]. The main difference between the CP-MAS ^13^C NMR spectra of cellulose and chitosan is the C-2 signal that is shifted to the higher field in chitosan (56 ppm) when compared to cellulose (73 ppm). Moreover, the signals corresponding to residual acetyl groups are observed in the CP-MAS ^13^C NMR spectrum of chitosan, i.e., the signals at 22 ppm (CH_3_) and 173 ppm (C=O), respectively. When chitosan was treated with sulfuric acid, the NH_2_-groups are converted to NH_3_^+^-groups and are ionically interacting with sulfonate ions. This resulted in broadening of all the signals observed in CP-MAS ^13^C NMR spectra and shift of C1- and C4-signals (supporting information).

The **70A,** seems to be a physical mixture of cellulose and chitosan with no changes in the chemical structure. While **70B** consisted of the low amount of chitosan, therefore based on this analysis, it is impossible to comment on possible changes in the chitosan part. The **70C** is a mixture of cellulose and ionically crosslinked chitosan. These observations are further supported by CP-MAS ^15^N NMR spectra (Figure 3b). The NH_2_-signal in unmodified chitosan resonates at 22.6 ppm, whereas the nitrogen atoms in the NHOAc-groups resonates at 121.7 ppm. When chitosan is treated with sulfuric acid, the nitrogen atoms in the corresponding NH_3_^+^- groups resonates at 32.0 ppm, which is regarded as a clear indication of the modification of chitosan (Supplementary Figure S2). 

In the **70C** and **70B**, the main signal in the CP-MAS ^15^N NMR spectra corresponds to the resonance frequency of NH_3_^+^- groups further strengthening the conclusion on ionic crosslinking of chitosan in these hydrogel beads. The corresponding signal in the CP-MAS ^15^N NMR spectra of **70A** is at 22.6 ppm, indicating that chitosan in this sample is not in ionic interaction with any counterion. Hence, the CP-MAS ^13^C and ^15^N NMR spectra support the results from IR and Raman spectroscopy.

### 3.4. XRD Analysis

**Figure 4:**
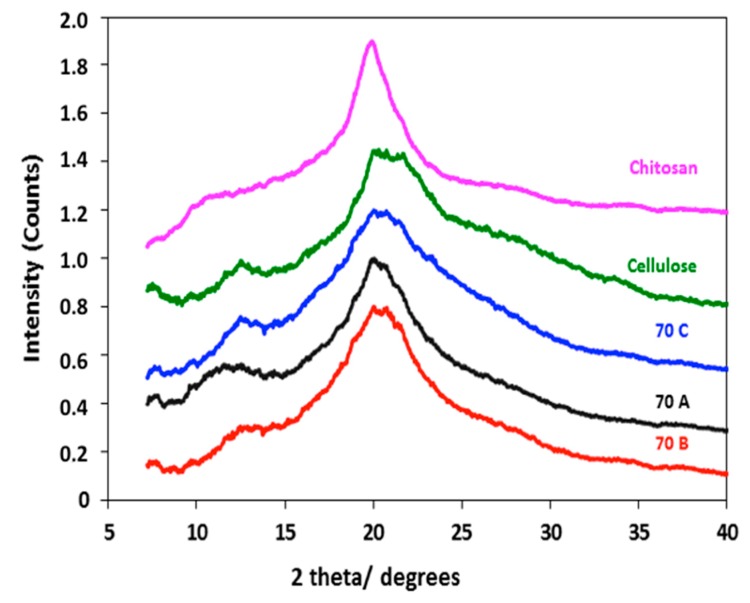
XRD diffraction patterns of chitosan, regenerated cellulose, **70A**, **70B** and **70C** chitosan-cellulose hydrogel beads.

The XRD analysis of pristine chitosan, regenerated cellulose, as well as **70A**, **70B**, and **70C** lyophilized hydrogel beads shows that chitosan has a different diffraction pattern when compared to the regenerated cellulose. In the case of pure chitosan, a sharp reflection at 2θ = 19° and a broad reflection at 10°–11° are present, while in cellulose broad reflections at 2θ = 22° and 12°–13° are observed. Since the beads are composites of chitosan and cellulose, an overlap of the chitosan (19°) and cellulose (22°) reflections in the beads is apparent. However, the relative peak intensities in the 10°–13° 2θ region can be used to distinguish the pattern of composite beads equivalent to chitosan or cellulose. The **70A** sample shows a pattern closer to the pure chitosan pattern, while **70B** and **70C** have patterns closer to cellulose. These observations could be explained by the fact that in **70A**, the chitosan has not undergone any changes during coagulation in 2 M acetic acid, while in **70B,** lower content of chitosan is present due to dissolution and leaching out from the hydrogel beads during gelation. In **70C**, the peak shapes resemble more the regenerated cellulose. This behaviour could be due to ionic interactions between chitosan and sulphuric acid. Therefore, the coagulating medium affects the crystallinity and the final composition of the beads, as shown in (Figure 4). 

### 3.5. Scanning Electron Microscopic Analysis (SEM) 

**Figure 5:**
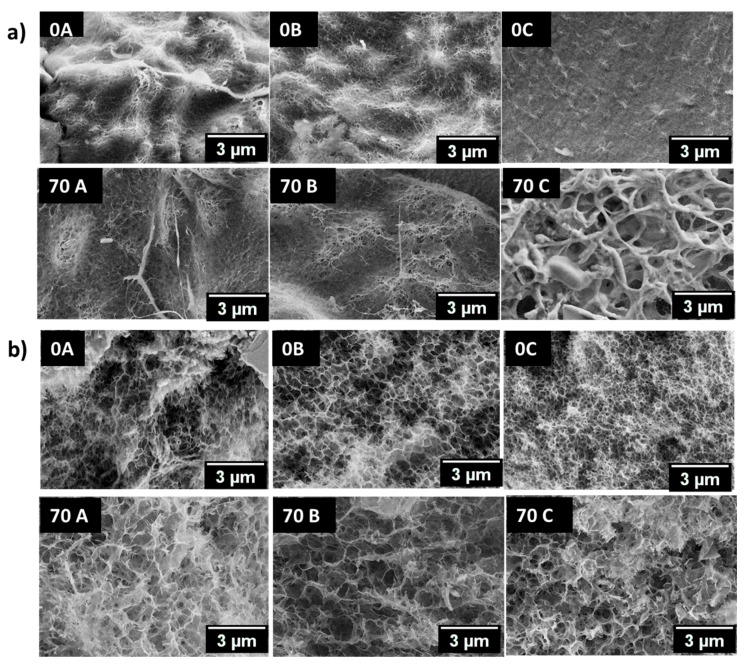
(**a**) Surface and (**b**) core morphology of **0A**, **0B**, **0C**, **70A**, **70B**, and **70C** chitosan–cellulose hydrogel beads with the scale bar 3 µm.

The qualitative morphological analysis of hydrogel beads was done by scanning electron microscopy (Figure 5). The pore size of the hydrogels is dependent on the polymer concentration, processing conditions, and the drying procedure. In the present study, liquid nitrogen was used to freeze the water in the hydrogel beads before the lyophilization. A considerable variation in the morphology and ultrastructure of all the samples was observed. A comparison of surface morphology of **0A**, **70A,** and **0B**, **70B** shows that the hydrogel beads have a porous surface with slightly larger pores in the case of **70A** and **70B**. In the case of **0C**, a highly compact ultrastructure is observed while the morphology is loose and porous for the **70C** sample. The cross-section ultrastructure evaluation of hydrogel beads has shown that **70A**, **70B,** and **70C** have increased porosity in comparison to the **0A**, **0B,** and **0C,** respectively. An unusual behaviour was observed in the hydrogel beads, namely fine fibrillary deposits were found on the surface of hydrogel beads in some regions. Thus, the variation in morphology of the hydrogel beads shows that the presence of chitosan and the coagulating medium governs the ultrastructure of the entities prepared.

### 3.6. Cytocompatibility Evaluation of Chitosan-Cellulose Hydrogel Beads with MDA-MB-231 Cells (Human Breast Adenocarcinoma—A Soft Tissue Organ)

**Figure 6:**
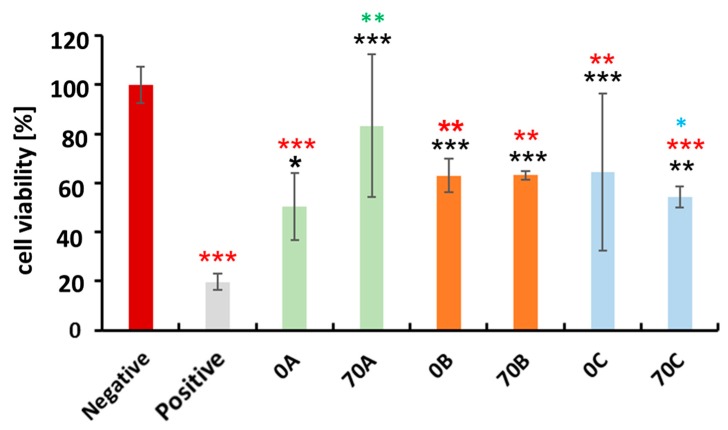
Human breast adenocarcinoma (MDA-MB-231) cytocompatibility with **0A**, **70A**, **0B**, **70B,** and **0C**, **70C** chitosan–cellulose hydrogel beads over the timeframe of 48 h. Values are expressed as percentage of the means ± SD (*n* = 4). Statistical significance was defined as * *p* < 0.05, ** *p* < 0.01, *** *p* < 0.005 compared to control samples (ANOVA test). Black * and red * indicates sample comparison to positive and negative control respectively. While green * shows comparison between **0A** and **70A**, **0B** and **70B**, **0C** and **70C**; blue * shows between **70A** and **70B**, **70A** and **70C**, **70B** and **70C**.

The human breast adenocarcinoma cells originate from the human female breast (a soft tissue organ), making them appropriate model cells for evaluating the potential of the developed materials for soft tissue engineering applications. For this purpose, we performed a cell viability testing. The cytocompatibility of the **70A**, **70B**, and **70C** hydrogel beads with MDA-MB-231 cells (human breast adenocarcinoma) over 48 h was evaluated using the WST-1 cell proliferation assay. A comparison between positive control and **0A**, **70A**, **0B**, **70B**, **0C**, **70C** was performed, as shown in (Figure 6). DMSO was used as positive control for cellular toxicity because at higher concentration it is toxic to the cells. As expected, all the groups showed higher cell proliferation as compared to DMSO. However, a comparison of all groups with negative control showed variable cytocompatibility amongst all of the hydrogel beads. The hydrogel beads **0A**, **0B**, **70B**, **0C**, and **70C** displayed less than 70% viability of the cells, which could be due to potential degradation products of the beads that could have eroded from the beads during their soaking. These could in turn partially hinder the growth of cells in the early stages of the experiment, resulting in lower viabilities when compared to the negative control. Thus further testing of these beads for any application is insignificant. Interestingly, a comparison between **0A**–**70A** showed that **70A** type has improved cytocompatiblity than the **0A** type, while we did not observe such difference between **0B**–**70B** and **0C**–**70C**. Further, between **70A**–**70B**, no significant difference was observed, but in case of **70A**–**70C**, the **70A** type was more cytocompatible than the **70C** type, possibly due to the presence of higher content of chitosan in the **70A** hydrogel beads where no counterions were present to interact with the amino groups, thus making them available to interact with the cells [2]. While in case of **70B** and **70C**, the amino groups are ionically crosslinked with the chloride and sulfonate or hydrogen sulphate groups, respectively, thus making amino groups unavailable to interact with the cells. Overall, **70A** type was comparatively more cytocompatible than the other hydrogel bead types with the model cells qualifying their use for soft tissue engineering. 

### 3.7. Cytocompatibility Evaluation of Chitosan-Cellulose Beads Coagulated in Acetic Acid with Osteoblast Cells (Hard Tissue)

**Figure 7:**
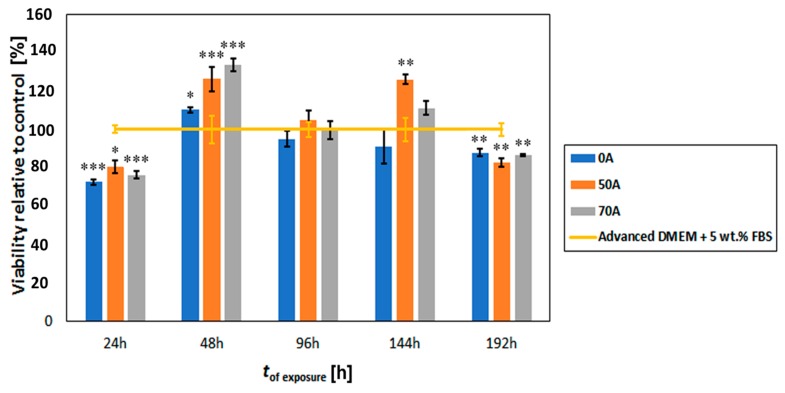
Osteoblast cytocompatibility (proliferation behaviour) with **0A, 50A** and **70A** chitosan–cellulose hydrogel beads based on the MTT assay over the timeframe of 192 h. Values are expressed as percentage of the means ± SD (*n* = 4). Statistical significance was defined as * *p* < 0.05, ** *p* < 0.005, *** *p* < 0.001 as compared to control sample (ANOVA test).

Proven cytocompatibility is the prerequisite for any scaffold to be used in bone tissue engineering. The main objective of this study was to evaluate the cytocompatibility of the chitosan^-^cellulose hydrogel beads coagulated in acetic acid with human bone-derived osteoblast cells, as shown in (Figure 7).

The results show a comparative cytocompatibility study between the control sample, i.e., Advanced DMEM, **0A**, **50A,** and **70A** at various times of exposure (up to 192 h). It is clear that the prepared beads affect the growth of the used cells, especially in comparison to the cell growth after each of the time intervals. After 24 h, we see a lower viability of the cells grown together with the bead groups, whereas already after 48 h, all of the prepared samples (**0A**, **50A**, and **70A**) show an increased viability, when compared to the control. Both samples that included chitosan (**50A** and **70A**) also showed a higher viability than the beads prepared from pure cellulose. Between them, the difference was in the range of rerpoted error for respective samples. After 96 h the viability was comparable to the control sample, whereas the samples **50A** and **70A** again outperformed the control sample after 144 h. Since at this time period, the sample **50A** showed the highest viability of all the samples, this might indicate that a modest number of free amino groups (from chitosan) has the most positive influence of the growth of osteoblast cells, whereas an even hugher number of free amino groups could potentially lead to a higher surface charge, which seems still suits the cells, but to a smaller extent. Regardless of the respective viabilities, these results definitely show that all of the tested (**0A**, **50A** and **70A**) beads are suitable for growth of osteoblast cells at least up to 144 h. 

After 192 h, we observed a decrease in the viability of the cells exposed to the bead samples, when compared to the control. Examination of the optical micrographs of the cells after the mentioned time period (not shown) showed no negative effect of the beads that could be observed in comparison with the control (no changes in cell morphology or shape etc.). It seems that the lower viabilities might be related to the fact that the cells have overgrown the experimental environment of the well in the P96 plate. In comparison with the control, where we do not see this decrease in viability, this effect is mainly due to the faster growth of cells in the case of sample exposure, potentially leading to contact inhibition. Further studies are of course necessary to confirm the latter via other methods, yet this is out of the scope of the present study.

A crystal violet dye viability/cytocompatibility assay was also performed to check the cytocompatibility of the hydrogel beads. The result is summarized in (Supplementary Figure S3). Finally, from Figure 7, we can also see that in the timeframe, where we observed an increased viability after exposure to the beads, in comparison with the control samples. The chitosan modified hydrogel bead outperform the pure cellulose beads, making these promising for future testing and development of such materials for bone tissue engineering applications.

Since our intention was not only to assess if the proposed hydrogel beads degrade in the cells native environment, which could hinder cell growth (Figure 7—cytocompatibility assessment), but also to evaluate the viability of the osteoblast cell culture directly on the as-prepared samples (attachment or direct contact test). However, we could not find any literature source that would describe an efficient method for direct visualization of grown cells on such hydrogel beads.

## 4. Conclusions

Cytocompatible chitosan-cellulose hydrogel beads were prepared with varying chitosan content and chemistry along the amino group. The hydrogel beads coagulated in 2 M acetic acid (**70A**) showed higher chitosan retention with free amino groups. Whereas, in case of a 2 M hydrochloric acid (**70B**), low amount of chitosan was present and the amino groups are ionically interacting with Cl^−^ ions. While in 2 M sulfuric acid (**70C**), although high chitosan was present, amino groups were involved in ionic interactions with HSO_4_^−^/SO_4_^2−^ ions. The cytocompatibility evaluation of the hydrogel bead groups with breast adenocarcinoma cell lines displayed **70A** type has the maximum cytocompatibility in comparison to other groups. Similarly, in the case of beads coagulated in 2 M acetic acid, the osteoblast cells showed effective adhesion to the bead surface, as well as an increased viability, when compared to the positive control. These results are presumably related to the form of amino groups present in the hydrogel beads. Concretely, the chitosan-cellulose hydrogel beads exhibiting free amino groups, led to a higher cell viability, when compared to the prepared samples, where the amino groups were involved in ionic interactions with the anions as in the case of Cl^−^ and HSO_4_^−^/SO_4_^2−^. Herein, we propose the chemistry and the mechanism behind the hydrogel beads coagulation in different acidic media, cytocompatibility with model soft and hard tissue cell lines and their future potential use in the bone tissue engineering applications.

## Supplementary Materials

The following are available online at www.mdpi.com/2306-5354/5/1/3/s1, Figure S1: SEM-EDS spectra of **70A**, **70B** and **70C** hydrogel beads, Figure S2: CP-MAS ^13^C and ^15^N NMR spectra of chitosan and chitosan treated with sulfuric acid, Figure S3: Crystal violet (CV) staining of beads with attached osteoblasts. Black spots and clusters show healthy cells (typical cells on all bead samples are marked with red circles). The darker the spots, the more viable the cells are (according to CV dyeing protocol). Control sample was dyed with CV for comparison as well.

Supplementary File 1
