# Supplementary material for: Chitosan–Cellulose Multifunctional Hydrogel Beads: Design, Characterization and Evaluation of Cytocompatibility with Breast Adenocarcinoma and Osteoblast Cells
Source: Bioengineering (Basel). 2018 Jan 9;5(1):3. doi: 10.3390/bioengineering5010003 (PMC5874869; doi:10.3390/bioengineering5010003)
Supplement: Supplementary File 1 [file bioengineering-05-00003-s001.pdf]

1. **SEM-EDS Results.**

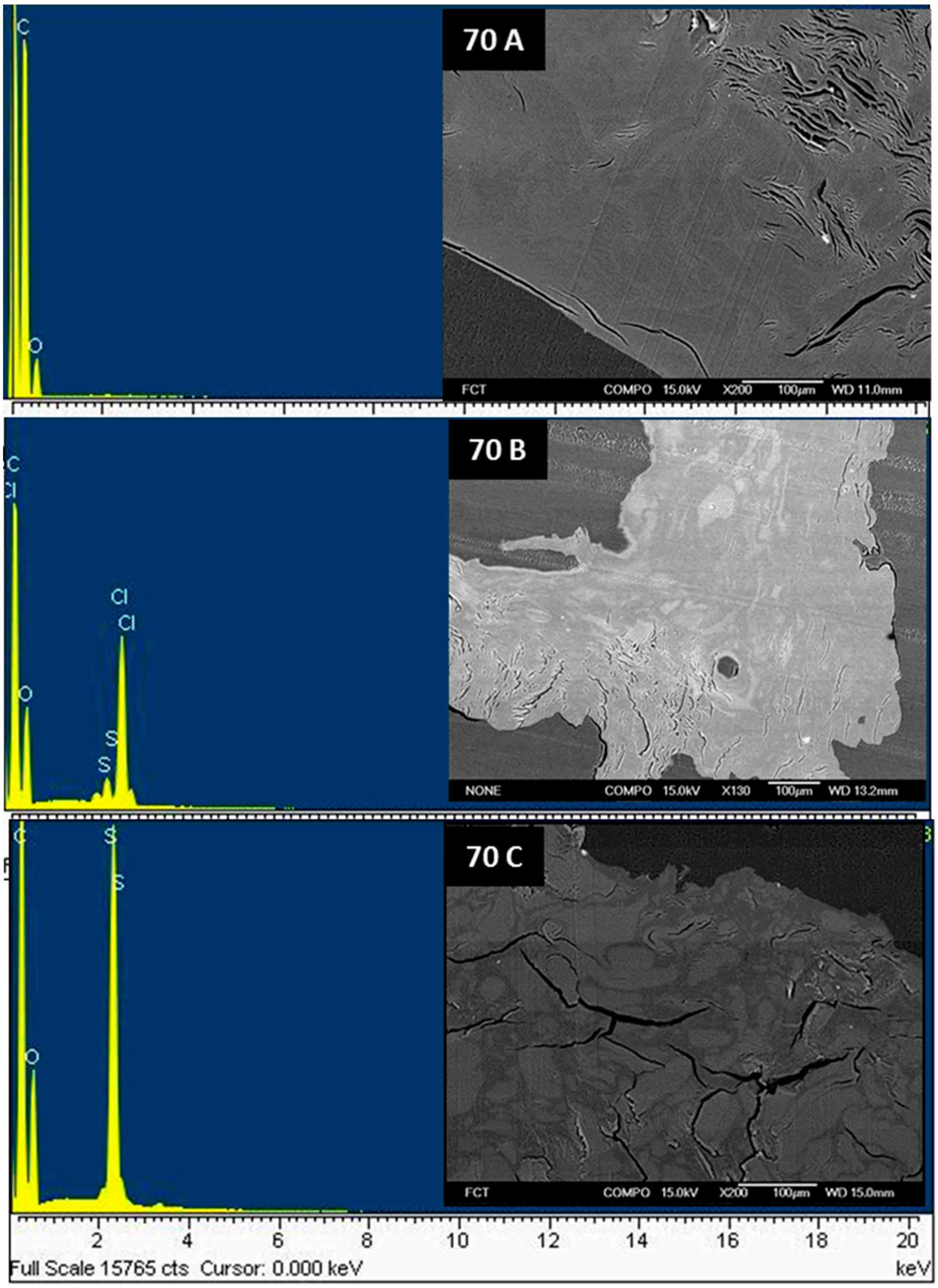

Figure. 1 SEM-EDS spectra of 70A, 70B and 70C hydrogel beads.

2. **NMR**

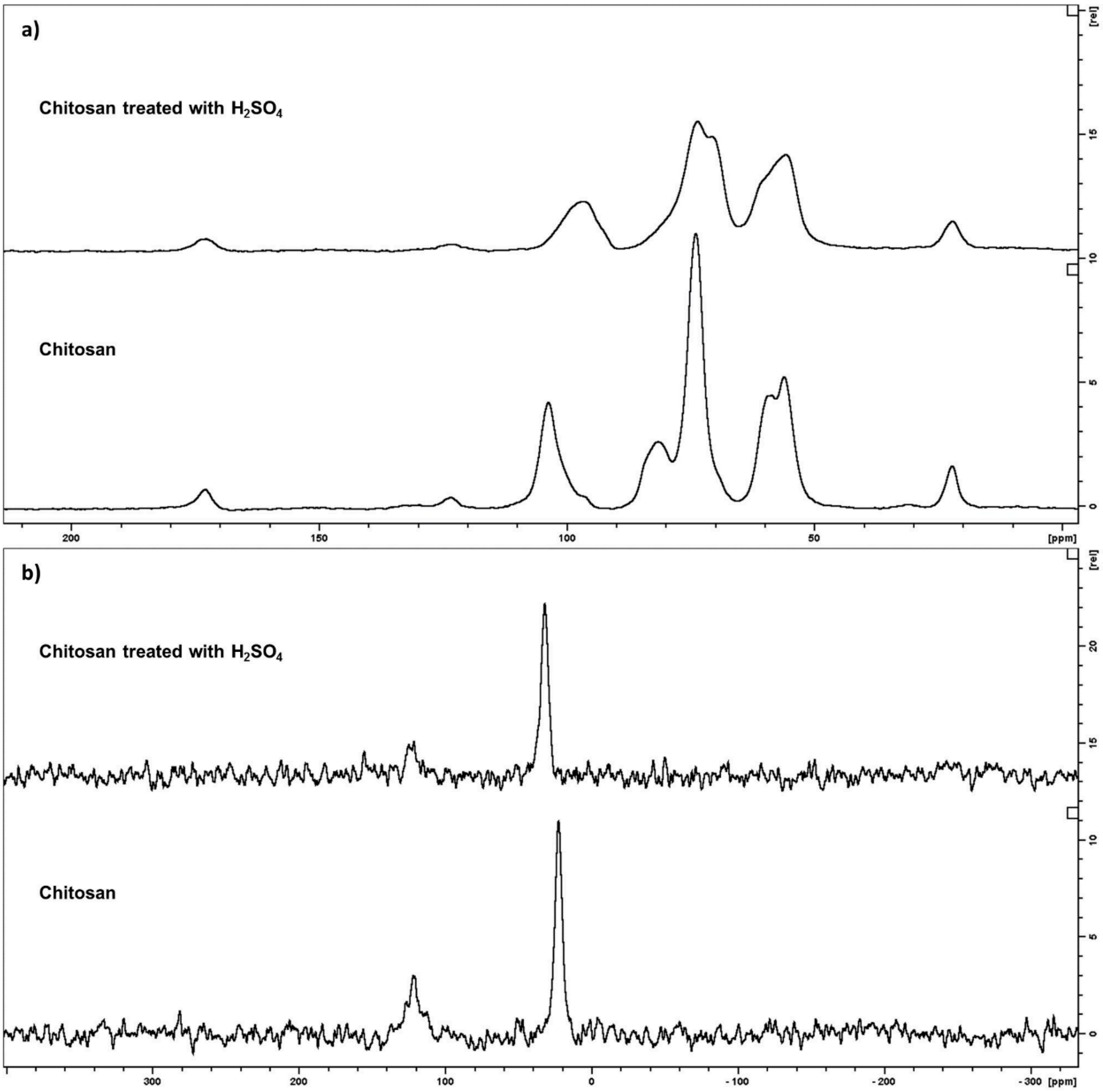

Figure2. CP-MAS ^{13}C and ^{15}N NMR spectra of chitosan and chitosan treated with sulfuric acid.

3. **Crystal voilet dye viability/biocompatibility assay**

A Live/Dead assay was used initially, but it seems that both dyes were rinsed from the bead surface during the protocol. Therefore, we used another commonly applied dye in viability/biocompatibility assessment, namely crystal violet (CV). Unfortunately, CV dyed also the hydrogel beads to some degree, but this did not prevent the visualization of clearly attached cells on the bead surface for all three sample types (**0A**, **70A** and **50A**). These results are shown in Figure 8 and again confirm the suitability of the

prepared hydrogel beads for further evaluation towards possible applications in bone (hard) tissue engineering.

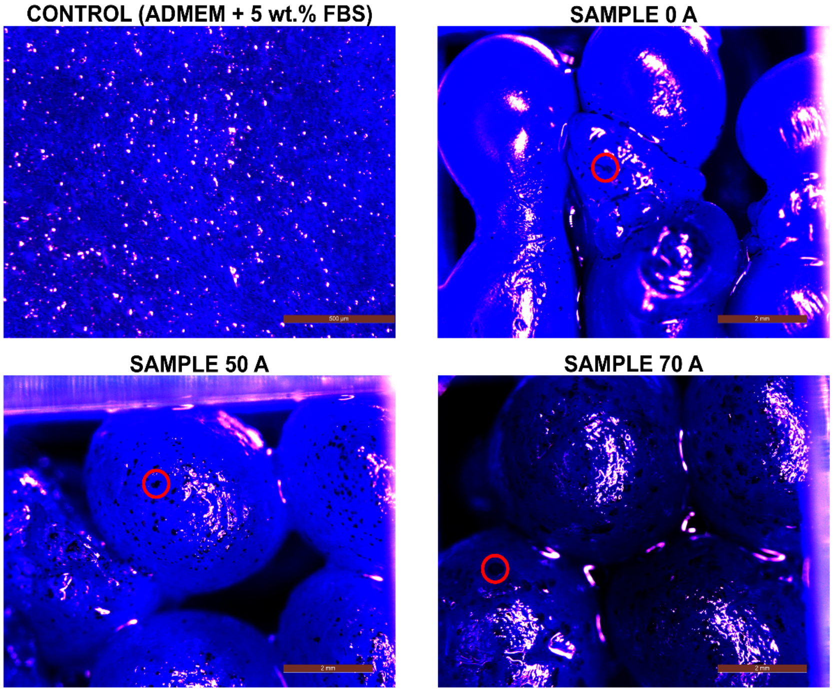

Figure 3. Crystal violet (CV) staining of beads with attached osteoblasts. Black spots and clusters show healthy cells (typical cells on all bead samples are marked with red circles). The darker the spots, the more viable the cells are (according to CV dyeing protocol). Control sample was dyed with CV for comparison as well
